# Curcumol Exerts Anticancer Effect in Cholangiocarcinoma Cells via Down-Regulating CDKL3

**DOI:** 10.3389/fphys.2018.00234

**Published:** 2018-03-20

**Authors:** Jinduo Zhang, Gang Su, Zengwei Tang, Li Wang, Wenkang Fu, Sheng Zhao, Yongjiang Ba, Bing Bai, Ping Yue, Yanyan Lin, Zhongtian Bai, Jinjing Hu, Wenbo Meng, Liang Qiao, Xun Li, Xiaodong Xie

**Affiliations:** ^1^The First Clinical Medical College, Lanzhou University, Lanzhou, China; ^2^Special Minimally Invasive Surgery, The First Hospital of Lanzhou University, Lanzhou, China; ^3^School of Basic Medical Sciences, Institute of Genetics, Lanzhou University, Lanzhou, China; ^4^Gansu Province Institute of Hepatopancreatobiliary, Lanzhou, China; ^5^Gansu Province Key Laboratory Biotherapy and Regenerative Medicine, Lanzhou, China; ^6^School of Stomatology, Lanzhou University, Lanzhou, China; ^7^The Second Department of General Surgery, The First Hospital of Lanzhou University, Lanzhou, China; ^8^Storr Liver Centre, Westmead Institute for Medical Research, University of Sydney, Westmead, NSW, Australia

**Keywords:** curcumol, proteomics, CDKL3, cell cycle, cholangiocarcinoma

## Abstract

Curcumol is the major component extracted from root of *Rhizoma Curcumae*. Recent studies have shown that curcumol exerts therapeutic effects against multiple conditions, particularly cancers. However, the therapeutic role and mechanism of curcumol against cholangiocarcinoma cells are still unclear. In our current research, we tested the effect of curcumol in cholangiocarcinoma cells, and using two-dimensional electrophoresis, proteomics and bioinformatics, we identified cyclin-dependent kinase like 3 (CDKL3) as a potential target for curcumol. We have demonstrated that curcumol can evidently suppress growth and migration of cholangiocarcinoma cells. Furthermore, curcumol could significantly block the cell cycle progression of the cholangiocarcinoma cells. These effects could be largely attributed to the inhibition of CDKL3 by curcumol. Further studies have recapitulated the oncogenic role of CDKL3 in that knockdown of CDKL3 by lentiviral mediated transfection of shRNA against CDKL3 also led to a significant inhibition on cell proliferation, migration, invasion, and cell cycle progression. Given the high level of CDKL3 expression in human cholangiocarcinoma tissues and cell lines, we speculated that CDKL3 may constitute a potential biological target for curcumol in cholangiocarcinoma.

## Introduction

Cholangiocarcinoma (CCA) is a fatal malignancy originated from biliary epithelial cells. According to its anatomical location, it is divided into intrahepatic (iCCA), perihilar (pCCA), and distal CCA (dCCA). Increased incidence of iCCA in Western countries has recently been reported (Patel, 2001; Siegel et al., 2016). The Age-adjusted incidence rate of iCCA in East Asia is highest in Khon Kaen of Thailand followed by Qidong of China (102.9/100,000 and 14.9/100,000, respectively) (Shin et al., 2010). CCA also has a high rate of recurrence and metastasis (Rahnemai-Azar et al., 2017), making this malignant tumor a serious clinical challenge. Over the past years, comprehensive therapy consisting of surgery, chemotherapy, and adjuvant radiation is regarded as the main treatment for CCA (Banales et al., 2016). However, CCA is generally insensitive to chemotherapy, making chemotherapy a less ideal approach (Rizvi et al., 2017). Based on the retrospective studies or small phase II trials, radiation therapy may be only beneficial for selected patients with liver-predominant disease (Lee and Cherqui, 2013). The success rate for surgical treatment is also less optimal, with the 5-year survival rate being 5–15% (Valle et al., 2017). Accordingly, more effective and safe treatments for CCA are critical and urgent.

Traditional Chinese Medicine has been increasingly used in cancer therapy (Wang et al., 2017). In fact, the herbal medicine *Rhizoma Curcumae* has been used for thousands of years in removing blood stasis and alleviating pain (Xia et al., 2005). *Rhizoma Curcumae* has been combined with another herbal medicine Astragali Radix in the treatment of cancers in Asian countries (Yin et al., 2018). Curcumol is a major component extracted from the root of the *Rhizoma Curcumae* (Lou et al., 2010), and has been shown to possess multiple therapeutic effects, such as antioxidant-, antimicrobial, anti-fibrotic, and anticancer activities (Dang et al., 2010; Yin et al., 2018). The anticancer effect of curcumol was reported to be related to its direct killing effect on tumor cells, along with inhibition of multiplication and induction of apoptosis (Tang et al., 2015). However, the effect of curcumol in the treatment of CCA and the underlying mechanisms is not clear yet. In view of the fact that curcumol has therapeutic potential for the treatment of gastrointestinal tumors, such as colon, gastric, and liver cancer (Wang et al., 2015; Zang et al., 2017), here we aimed to investigate the impact of curcumol on CCA cells and clarify the possible molecular mechanisms. Based on our proteomic studies and bioinformatic analysis, we identified that cyclin-dependent kinase like 3 (CDKL3), also known as NKIAMRE, is likely involved in the development of CCA. CDKL3 has a similar sequence with cyclin-dependent kinase 3 (CDK3) (Zheng et al., 2017). CDKL3 contains two highly conserved sequences that are present in mitogen-activated protein kinases or cyclin-dependent kinases (Yee et al., 2003). Previous studies have revealed that overexpression of CDKL3 was present in the invation anaplastic large cell lymphoma, and up-regulation of CDKL3 was reported to enhance cell proliferation of various mammalian cell lines, promote the transition from G0/G1 stage to S stage and accelerate cells enter the DNA synthesis stage phase (Thompson et al., 2005; Jaluria et al., 2007).

The results of our study proved that CDKL3 may function as an oncogene in CCA, and curcumol may exert tumoricidal effect against CCA through down-regulating CDKL3.

## Methods

### Materials

Curcumol and dimethyl sulfoxide (DMSO) were obtained from Sigma-Aldrich (MO, USA). Cell Counting Kit-8 (CCK8) was obtained from Dojindo (Kumamoto, Japan). Annexin V-FITC Apoptosis Detection Kit and Annexin V-APC Apoptosis Detection Kit were purchased from eBioscience (Hatfield, UK). The Cell Cycle Analysis Kit was obtained from Wanlei (Changchun, China). Rabbit anti-CDKL3 antibody was from obtained from Proteintech (Chicago, USA); anti-β-actin antibody was obtained from Abcam (Cambridge, UK). Complementary oligonucleotides containing a short hairpin RNA (shRNA) targeting CDKL3 were dimerized and cloned into the pFU-GW lentiviral vector by Genechem (Shanghai, China).

### Cell culture

Two CCA cell lines, RBE (purchased from Genechem, Shanghai, China) and HCCC-9810 (purchased from Procell Life Science&Technology Co.,Ltd. Wuhan, China) and human intrahepatic biliary epithelial cells (HIBEC, purchased from Procell Life Science&Technology Co.,Ltd. Wuhan, China) were used in this work. These Cells were cultured according to the manufacturer's instructions. Curcumol was dissolved in DMSO to a stock concentration of 20 mg/ml. In subsequent experiments, the stock curcumol was diluted in RPMI 1640 medium for all treatments. The concentration of DMSO was kept to <1% in all conditions.

### Proliferation assay

The effect of curcumol on proliferation of CCA cells was measured by CCK8 assay. In a nutshell, cells were cultured in a 96 well plate, each well containing 4 × 10^3^cells and incubated for 12 h. Cells were treated with different concentration of curcumol (50, 60, 75, 100 μg/mL). After 48 h, 10 μL/well CCK8 was added and then incubated for another 2 h. The plates were read at 450 nm on a TECH M200 Plate Reader (TECH, Switzerland). The Cell viability was calculated by adjusting the control group (culture medium containing 1% DMSO) to 100%, and all treatment groups normalized against the adjusted control group. All experiments were performed three times.

### Migration assay

Scratch assay was used to examine the ability of CCA cells to migration after treatments. Cells were inoculated on 6-well plate and grown to confluence. A 200-μl tip was used to make a denuded area (0 h). Cells were flushed with phosphate buffered saline (PBS) for two times and cultured with different curcumol (75, and 100 μg/mL). Migration was monitored under the BDS200 Inverted Biological Microscope (Optec, Chongqing, China) and photos were taken at 0, 24, 48, and 72 h. Cell migration distance was expressed as fold change over the control. All experiments were performed three times.

### Cell cycle assay

Cell cycle distribution was detected by flow cytometry (FCM) as follows. After the curcumol (75 and 100 μg/mL) treatment for 48 h, collected cells and flushed with PBS twice, then fixed in 70% ice-cold ethanol overnight. Then washed cells with cold PBS twice and adjusted to a concentration of 1 × 10^6^/ ml/ well, incubated with 100 μL RNase A for 30 min at 37°C, and then stained with 500 μL propidium iodide away from light at room temperature for 30 min. Cells were analyzed by FCM (Becton Dickinson, San Jose, CA, USA).

### Detection of apoptosis

Apoptosis was assessed by FCM using Annexin V-FITC /PI or Annexin V-APC staining. Briefly, after treatment with curcumol (75 and 100 μg/mL) for 48 h, washed cells twice with PBS, centrifugated at 1500 rpm for 5 min, stained with Annexin V-FITC /PI or Annexin V-APC, then analyzed byFCM (Becton Dickinson, San Jose, CA, USA).

### Two-dimensional gel electrophoresis (2-DE) and MALDI-TOF/TOF mass spectrometry analysis

Briefly, RBE cells were cultured with 100 μg/ml curcumol for 48 h. Cells treated with 1% DMSO was used as controls. Washed cells twice with cold PBS, incubated for overnight and exposed to Lysis Buffer B (7 M urea, 2 M thiourea, 4%/v CHAPS, 1% w/v DTT, 0.5% Ampholyte and a cocktail of protease inhibitors). Cells were placed on ice for 30 min and then centrifugated at 12,000 rpm for 30 min at 4°C. The supernatant was collected for 2D-PAGE analysis. Protein concentration was measured by the Bradford Assay (Tiangen, Beijing, China). The isoelectric focusing (IEF) was performed with Bio-Rad PROTEAN IEF Cell according to the manufacturer's instructions. In brief, 70 μg of cellular protein was loaded onto an IPG strip N (24 cm, pH 3.0–10.0, linear gradient). After 12 h, the strips were rehydrated for 24 h, and the proteins were separated by a second dimensional separation (SDS-PAGE) in 1.0 mm-thick 10% polyacrylamide gels in a Bio-Rad PROTEAN II XI Cell System (Bio-Rad, California, USA). The proteins resolved in the gel were visualized by the modified silver stain, scanned with Bio-Rad VersaDoc Imaging System, and analyzed using PDQuest 8.0.1 Software (Bio-Rad, California, USA). The selected protein spots were analyzed with ABI 5800 Plus MALDI-TOF/TOF Spectrometer (Applied Biosystems, California, USA) after in-gel digestion. Peptide mass mapping was conducted using the MASCOT V2.1 search engine (Matrix Science, London, UK) against the NCBInr protein databases with a GPS explorer software, V3.6 (Applied Biosystems, California, USA). Proteins were identified based on the minimum number of matched peptides. Each experiment was performed in triplicate.

### Immunohistochemistry assay

CCA and congenital choledochal cyst (CCC) tissue sections of paraffin-embedded samples were collected from 40 patients (20 CCA samples and 20 CCC samples) who admitted to the First hospital of Lanzhou University (Lanzhou, China) during January 2010 to December 2017. Routine immunohistochemical staining was performed to detect the tissue expression of CDKL3. Briefly, 5-μm tissue sections were mounted on the Ultra-Thin Semiautomatic Microtome (Thermo Fisher Scientific, Waltham, MA, USA), deparaffinized and rehydrated through graded alcohol rinses. Pressure cooker antigen retrieval was performed by ethylene diamine tetraacetic acid (EDTA) for 2 min after boiling. Endogenous peroxidase activity quenched using 0.3% H_2_O_2_ for 10 min and non-specific staining of slides was blocked with 3% BSA for 1 h. Paraffitn-embedded tissue sections were incubated with the rabbit anti-human CDKL3 (diluted at 1:200) at 4°C overnight, washed for three times with TBS containing 0.1% of Tween-20 (5 min each), then incubated with MaxVision™ HRP-Polymer anti-Mouse/Rabbit IHC Kit (MaxVision, Fuzhou, China) at room temperature for 1 h. Color development was done using DAB+ Chromogen, (MaxVision, Fuzhou, China). Hematoxylin was utilized for counterstaining.

The study was approved by the Ethics Committee for Human Research, Lanzhou University, and written informed consents were obtained from all patients.

### Knockdown of CDKL3

RBE Cells were seeded in 6-well plate and culture for 12 h until they grew to 20–30% confluency prior to transfection. The siRNAs (MOI 20) and polybrene (5 μg/ml) were complexed in RPMI 1640 medium. The siRNA-treated cells were additionally maintained for 72 h to achieve a complete transfection. The knockdown efficiency was confirm using quantitative real-time PCR (qRT-PCR) and Western blot analysis. Each experiment was performed in triplicate.

### Proliferation, migration, and invasion assays

The impact of CDKL3 on proliferation and migration of CCA cells was examined in the stable cells by CCK8 assay and scratch assay, as described above. The impact of CDKL3 on the invasion of CCA cells was examined by transwell assays. Briefly, 24-well transwells pre-coated with Matrigel (BD Biosciences, Franklin Lakes, NJ) were used. 1 × 10^5^ cells were suspended in 200 μL RPMI 1640 medium without FBS were added to the upper chamber. 800 μL RPMI 1640 with 30% FBS was added to the lower chamber. After 48 h the Matrigel and cells remained in the upper chamber were removed using cotton swabs. Cells on the lower surface of the membrane were fixed in 4% paraformaldehyde and stained with crystal violet. Cells were counted under the microscope in five microscopic fields and photographed (200 × magnification). Each experiment was performed in triplicate.

### Cell cycle and apoptosis assays

The impact of CDKL3 on cell cycle progression and apoptosis was further analyzed by FCM using the Cell Cycle Analysis Kit and Annexin V-APC stain, as described above. Each experiment was performed in triplicate.

### qRT-PCR analysis

Thirty one CCA samples and matched non-tumorous tissues (snap-frozen tissues) used for qRT-PCR were consecutively collected from patients who underwent curative resection between January 2010 to December 2017 at the same department. Briefly, total RNA was extracted from the tissues and cells using TRIzol reagent (SANGON), according to the instructions. Total RNA (1 μg) was reverse-transcribed into cDNA using PrimeScript™RT reagent kit with gDNA Eraser (Takara, Dalian, China), and qRT-qPCR was performed with SYBR Premix Ex Taq II (Takara, Dalian, China). Specificity of PCR product was confirmed by melting point analysis and the relative fold change in gene expression was analyzed as 2^−ΔΔCT^.

The primers used in qRT-qPCR were obtained from Sangon (Shanghai, China), and the sequences are as follows (F: forward; R: reverse).

CDKL3_F: AAAGTGGGCAATTTGTCACCT;
CDKL3_R: TTGGGGTGTTGAACTTGAGGA;
CyclinD1_F: AAAGTGGGCAATTTGTCACCT;
CyclinD1_R: TTGGGGTGTTGAACTTGAGGA;
CDK4_F: CTGGTGTTTGAGCATGTAGACC;
CDK4_R: AAACTGGCGCATCAGATCCTT;
P21_F: GCAGACCAGCATGACAGATTTC;
P21_R: CTTCCTGTGGGCGGATTAGG;
P27_F: CATAGATGCCGCGGAAGGT;
P27_R: CTGCAACCGACGATTCTTCTACT;
GAPDH_F: TGACTTCAACAGCGACACCCA;
GAPDH_R: CACCCTGTTGCTGTAGCCAAA.

### Immunoblot analysis

Harvested cells were washed twice with PBS twice and exposed to RIPA lysis with 1 mM PMSF. Cell lysates were placed on ice for 30 min to facilitate protein extraction, and then centrifuged at 12,000 rpm for 30 min at 4°C. The protein concentration was quantified using BCA protein assay kit. Equal amount of protein (40 μg/lane) was loaded onto a 10% sodium dodecylsulphate polyacrylamide (SDS-PAGE) gel for electrophoresis, and the proteins were transferred onto a Nitrocellulose membrane. Non-specific binding sites were blocked with 3% BSA for 1 h followed by incubation with appropriate primary antibody at 4°C overnight. The blots were subsequently washed with TBST for three times each for 5 min, and then probed with secondary antibody at room temperature for 1 h. Then washing with TBST for 3 times each for 5 min, the signals were visualized with enhanced chemiluminescence kits (BIO-RAD, USA).

### Statistical analyses

Data are expressed as means ± Sd. Difference between the mean values of each group was assessed by the Student's *t* test. Difference was considered statistically significant when a *P*-value is <0.05. All experiments were performed at least three times and representative results are shown.

## Results

### Curcumol inhibited cell proliferation and migration

**Figure 1:**
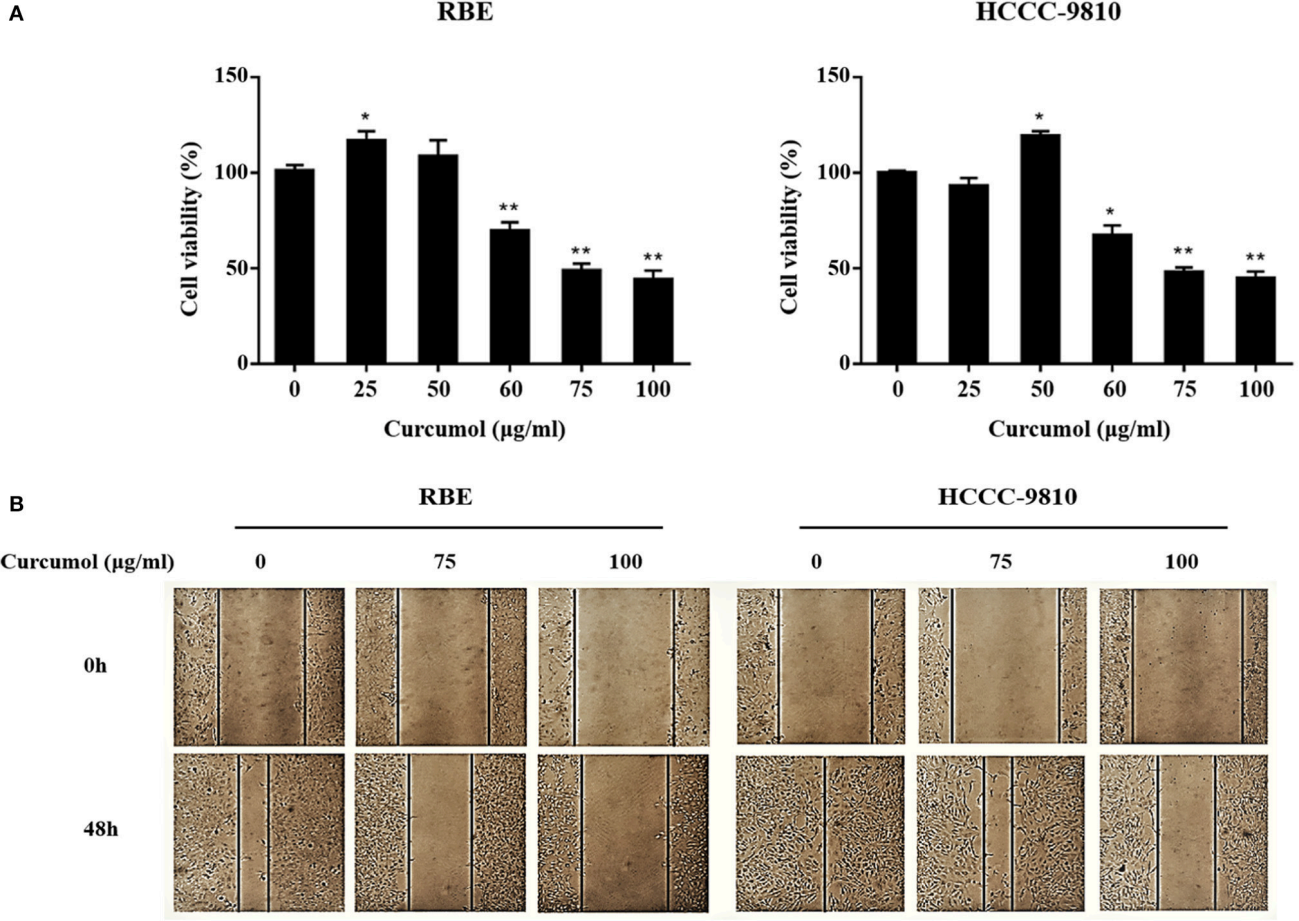
Curcumol inhibits proliferation and migration of CCA cells. Viability of curcumol-treated RBE and HCCC-9810 cells was evaluated by CCK8 assay **(A)**. Effect of curcumol on the migration of RBE and HCCC-9810 cells by scratch assay. Representative images were captured at 48 h after treatment. Data represent mean ± SD from at least three independent experiments **(B)**. *P < 0.05, **P < 0.01, vs. control cells.

To determine the activity of curcumol on CCA cells, we tested if curcumol shows any inhibitory effect on the proliferation of CCA cells first. Using CCK8 assay, we observed that curcumol did not affect the viability of RBE and HCCC-9810 cells at the does of <50 μg/ml (Figure 1A). At the higher doses, curcumol showed a does-dependent cytotoxic effect against RBE and HCCC-9810 cells (Figure 1A).

Curcumol was also observed to inhibit the migratory ability of the RBE and HCCC-9810 cells, as shown in Figure 1B, where treatment of the cells by curcumol (75 and 100 μg/mL, for 48 h) clearly inhibited cell migration.

### Curcumol block cell cycle progression at G1 stage and induces apoptosis

**Figure 2:**
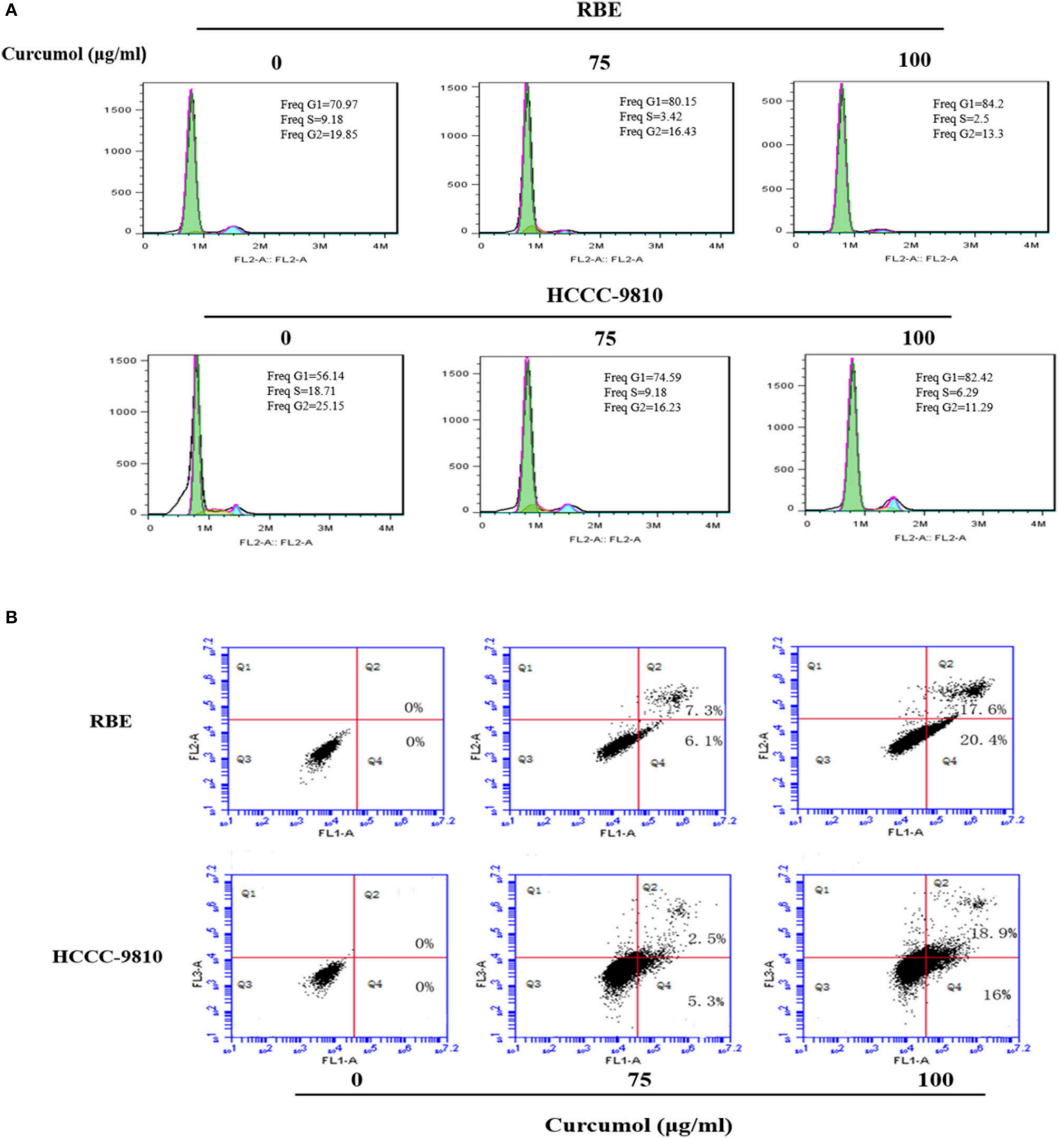
Curcumol blocks cell cycle progression at G1 phase and induces apoptosis in CCA cells. **(A)** Curcumol induced G1/S cell cycle arrest in RBE and HCCC-9810 cells. Representative flow cytometry images at 48 h of treatment are shown. **(B)** Curcumol induced apoptosis in RBE and HCCC-9810 cells following treatment with curcumol for 48 h. Representative dot plots are shown.

Cell cycle arrest is a common mechanism for many anticancer agents (Maes et al., 2017). Here, we demonstrated that curcumol increased the percentage of cells at G0/G1 stage (Figure 2A). In order to determine whether the inhibitory effect of curcumol on growth of CCA cells is related to induction of apoptosis, we calculated the percentage of RBE and HCCC-9810 cells undergoing apoptosis following curcumol treatment by Annexin V-FITC /PI fluorescence staining assay (Figure 2B), curcumol (75 and 100 μg/mL, for 48 h) induced significant apoptosis in RBE and HCCC-9810 cells.

### Effect of curcumol on G1 phase related genes

**Figure 3:**
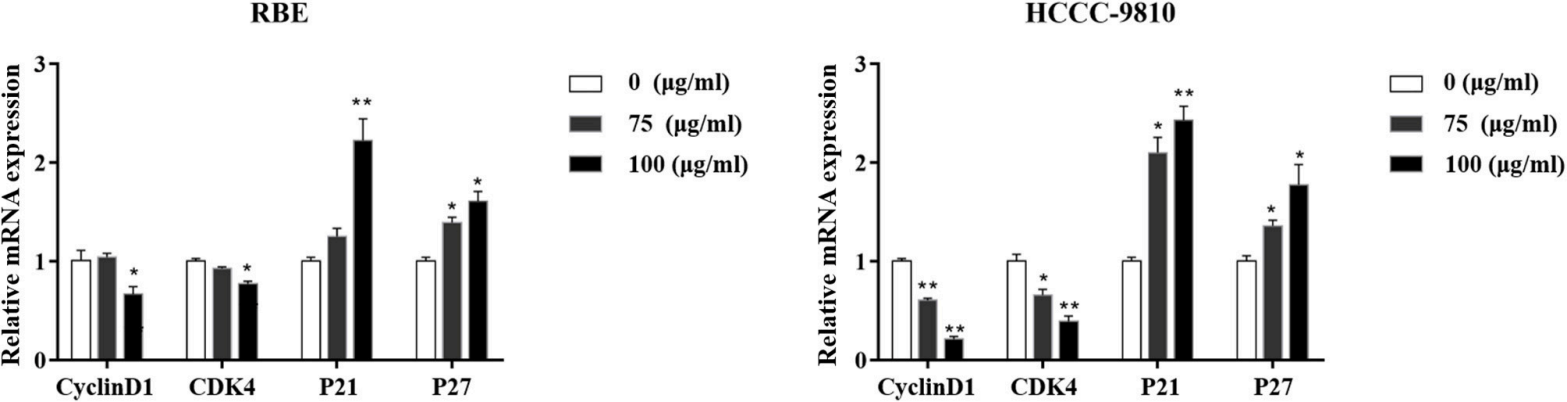
Effect of curcumol on the expression of the cell cycle associated molecules in RBE and HCCC-9810 cells. Curcumol decreased the mRNA level of cyclinD1 and CDK4, while it increased the expression of P21 and P27. *P < 0.05, **P < 0.01, vs. control cells. GAPDH was used as internal controls.

The above data demonstrated that curcumol could significantly affect the proliferation and cell cycle progression. We then try to confirm the impact of curcumol on the expression of cell cycle regulatory genes such as p21, P27, cyclinD1, and CDK4, which are vital on transition of cell cycle from G1 to S stage (Milewski et al., 2017). RBE and HCCC-9810 cells were pre-treated by curcumol (75 and 100 μg/mL, 48 h), and the expression level of the above genes was determine at the mRNA level by qRT-PCR. As shown in Figure 3, curcumol markedly decreased the mRNA levels of cyclinD1 and CDK4, while the mRNA expression of P21 and P27 was significantly increased. These data indicated that curcumol-induced cell cycle arrest is correlated with its inhibitory effect on G1 Phase genes in CCA cells.

### Identification of the proteins mostly affected by curcumol in RBE cells by proteomics

**Figure 4:**
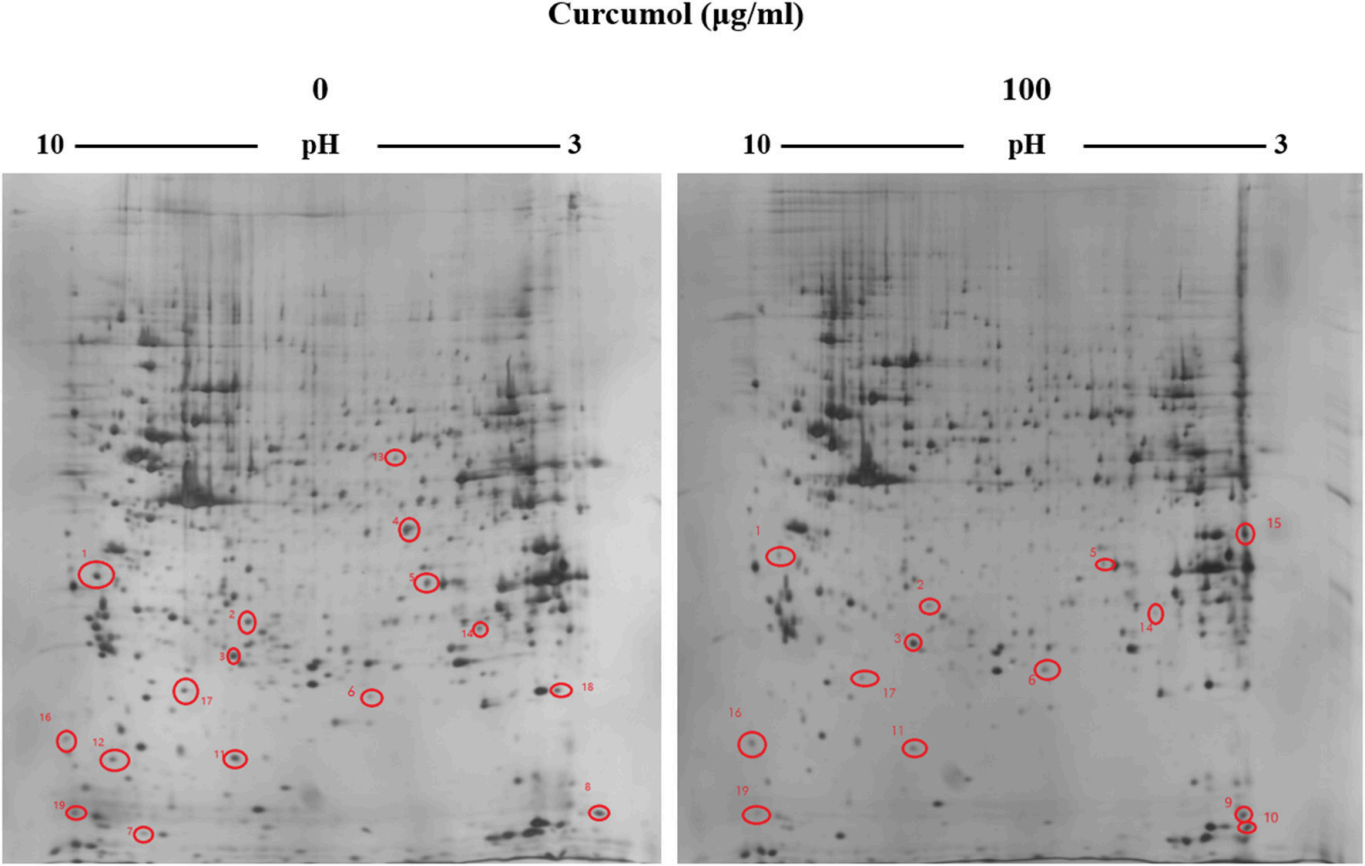
Representative 2-DE gels derived from the RBE cells exposed to curcumol or 1% DMSO. Cells were treated with 100 μg/ml of curcumol or 1% DMSO for 48 h. The differentially expressed protein spots were marked on the maps. The map was a representative of three independent runs.

To search for the potential molecular targets underlying the anti-tumor effect of curcumol, we compared the protein expression patterns between control and the RBE cells treated with 100 μg/ml curcumol for 48 h. Gels with marked protein spots were used for the analysis. Using 1.2-fold change as the cut off, we identified 19 spots as the most significantly altered proteins following curcumol treatment (Figure 4). Among the 19 differentially expressed proteins, 15 were down-regulated and 4 were up-regulated, all with an average of 2-fold or greater changes (*P* < 0.05). These spots were isolated and digested with trypsin. The identification of these proteins was examined by MALDI-TOF-MS. Eventually, three proteins were successfully identified. The relevant information for each protein is summarized in Table 1.

**Table 1:** Identification of the protein spots in RBE cells exposed to curcumol by MALDI-TOF-MS.

**Protein name**	**Gene name**	**Accession No**.	**Calculated Mr**	**Mascot score**
Heat shock protein β-1	HSPB1_HUMAN	P04792	22826	264
ATP synthase subunit alpha, mitochondria precursor	ATP5A1_HUMAN	P25705	56265	329
NKIAMRE	CDKL3_HUMAN	Q8IVW4	52000	235

### Expression of CDKL3 in human CCA tissues and established cell lines

Based on the proteomics analysis, we identified that CDKL3 is likely an important molecule in CCA. Previously published studies have demonstrated that curcumol could inhibit the proliferation, migration, and cell cycle of colon cancer cells (Wang et al., 2015, 2018). Similarly in our study, curcumol possesses anti-proliferative effect in CCA cells. As CDKL3 was previously shown an important role in the proliferation and cell cycle regulation in HeLa, HEK-293, CHO and MDCK cells (Yee et al., 2003; Jaluria et al., 2007), we sought to verify whether the anticancer effect of curcumol in CCA cells is also mediated through CDKL3.

**Figure 5:**
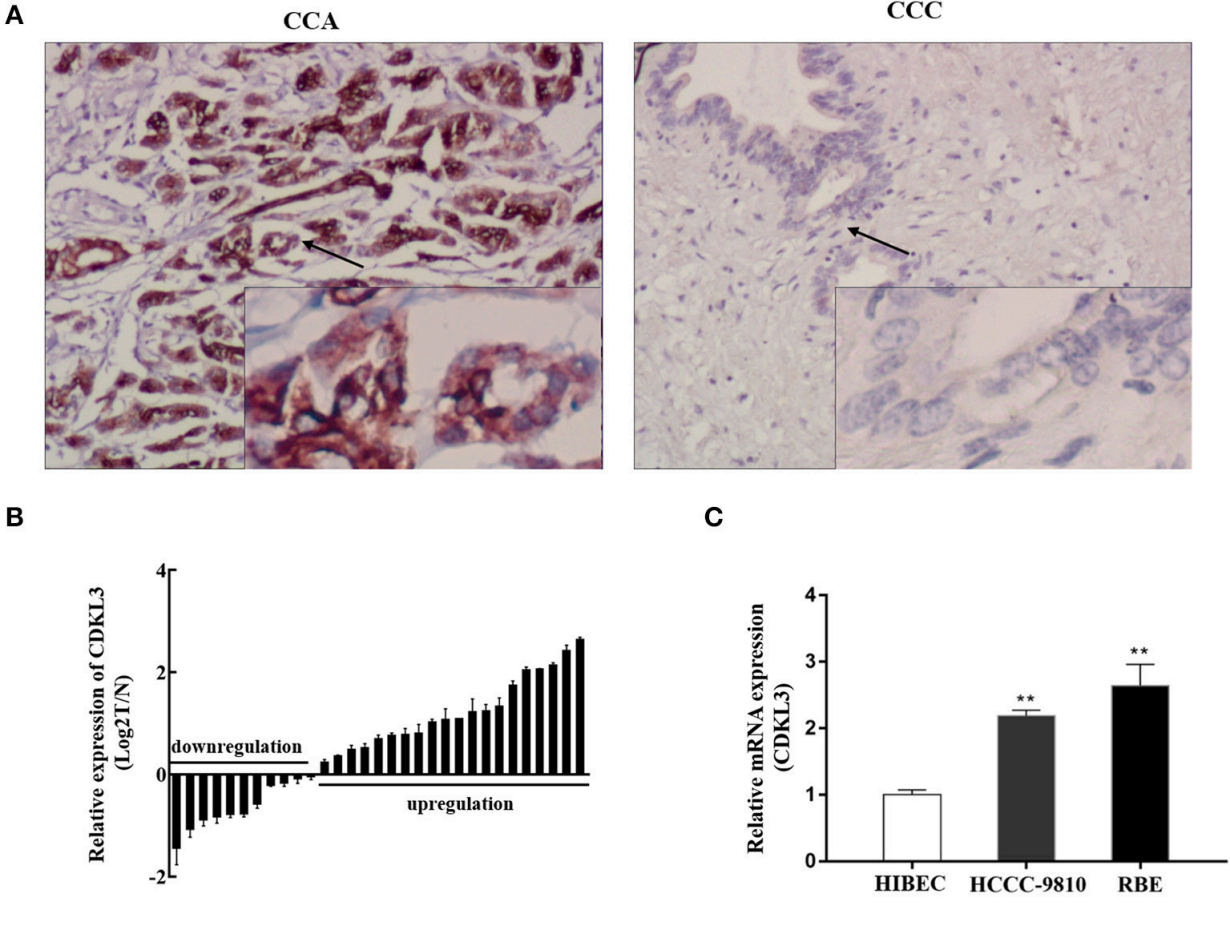
Expression of CDKL3 in human CCA tissues and established cell lines. **(A)** Expression of CDKl3 in human CCA tissues and control tissues was detected by immunohistochemistry. **(B)** Expression of CDKL3 in 31 pairs of tumor samples and matched adjacent noncancerous tissues was examined by qRT-PCR. **(C)** Expression of CDKL3 in human intrahepatic biliary epithelial cells (HIBECs) and 2 human cholangiocarcinoma cell lines (RBE and HCCC-9810) was examined by qRT-PCR. *P < 0.05, **P < 0.01, vs. HIBEC cells.

The results of immunohistochemistry (Figure 5A) and qRT-PCR analyses (Figure 5B) showed significantly higher CDKL3 levels in CCA tissues. In the CCA tissues, positive CDKL3 was mostly present in the cytoplasm of tumor cells (Figure 5A). The expression of CDKL3 was also examined in a group of CCA cell lines by qRT-PCR. As shown in Figure 5C, two CCA cell lines RBE and HCCC-9810 express relatively higher level of CDKL3 in comparison with HIBECs. Hence, high expression of CDKL3 is present in CCA tissues and cells.

### Curcumol significantly inhibited the expression of CDKL3 in CCA cells

**Figure 6:**
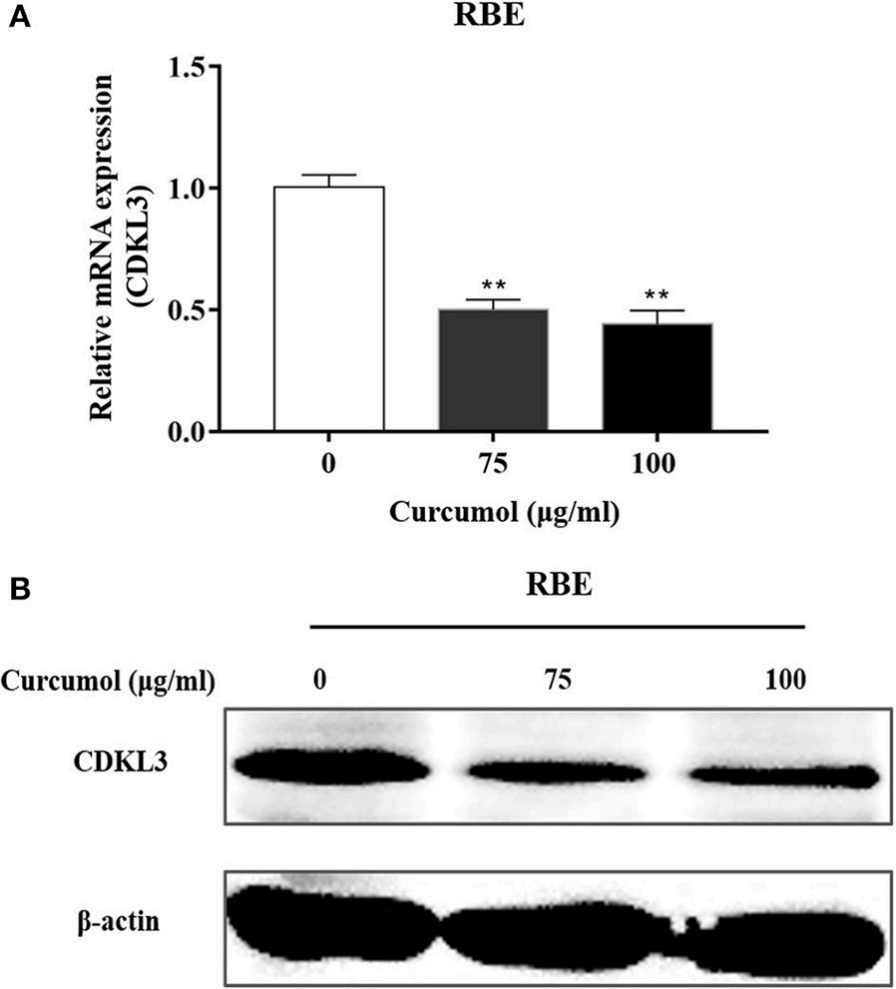
Effect of curcumol on the expression of CDKL3. Expression of CDKL3 in RBE cells treated with curcumol (75 and 100 μg/ml, 48 h) was detected by qRT-PCR **(A)** and Western blotting **(B)**. The results shown are representative of three independent experiments. *P < 0.05, **P < 0.01, vs. control cells.

As Figure 6 shows, treatment of RBE cells with 100 μg/ml curcumol significantly reduced the expression of CKLD3 at mRNA (**A**) and protein levels (**B**).

### Knockdown of CDKL3 inhibits proliferation, migration, and invasion of RBE cells

**Figure 7:**
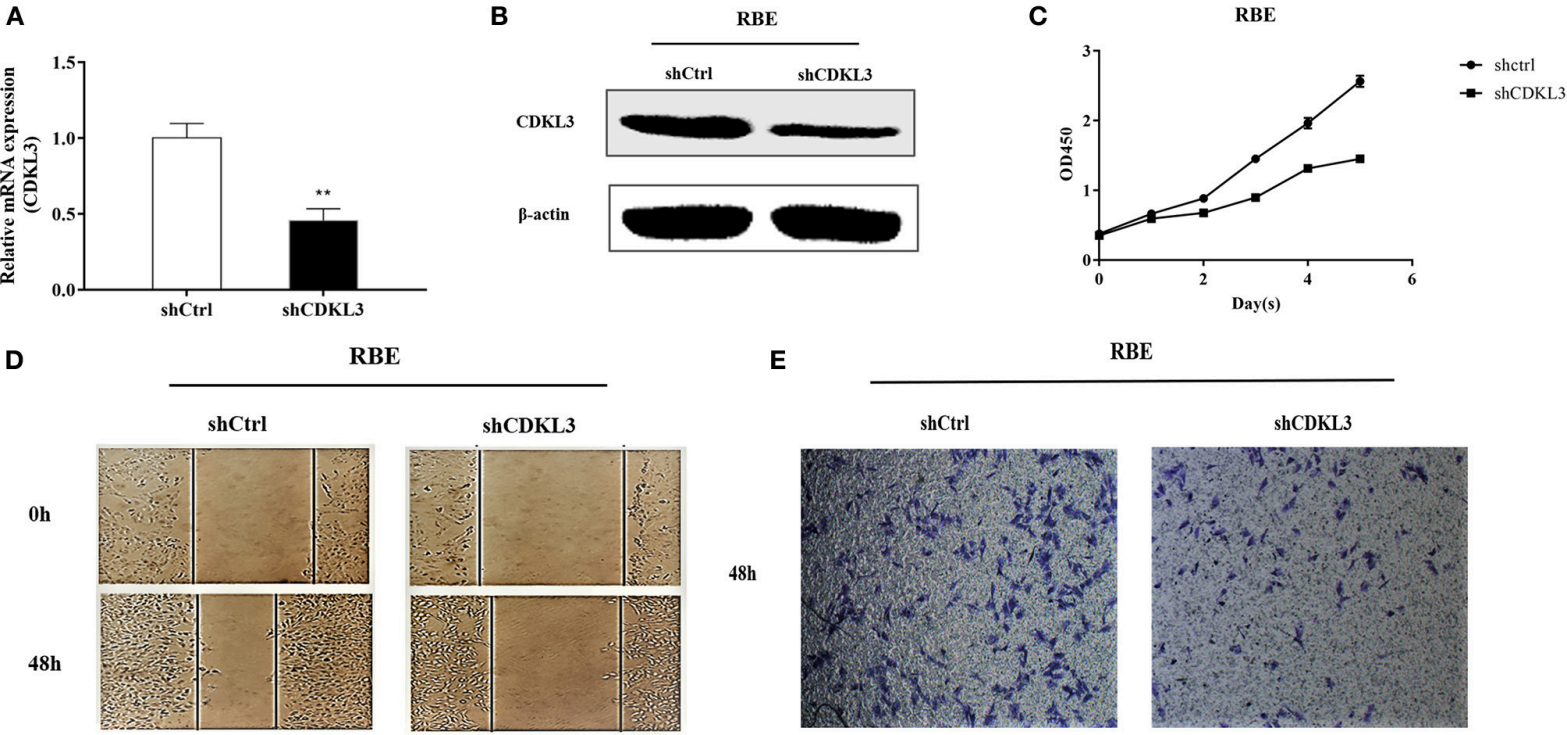
Knockdown of CDKL3 inhibits proliferation, migration and invasion of RBE cell. Representative RT-PCR **(A)** and Western blot analysis **(B)** of CDKL3 knockdown by lentiviral shRNA against CDKL3 are shown. Knockdown of CDKL3 greatly inhibited the proliferation **(C)**, migration **(D)**, and invasion **(E)** of RBE cells. Values represent mean ± SD. *P < 0.05, **P < 0.01, as compared with shCtrl.

The above results suggested that curcumol possesses an antitumour effect against CCA, and such a suppressive effect may be attributed to the inhibitory effect of curcumol on CDKL3. To further verify if curcumol inhibits the proliferation, migration, and invasion of the CCA cells through CDKl3 signaling, we knocked down CDKL3 by using its specific shRNA. Thus, RBE cells were transfected with lentiviral vector encoding shCDKL3 or shCtrl, and the subsequent biological consequences were examined. As shown in Figure 7, knockdown of CDKL3 (**A**,**B**) led to a significant inhibition of cell proliferation (**C**), migration (**D**), and invasion (**E**).

### Knockdown of CDKL3 blocks cell cycle progression at G1 phase

**Figure 8:**
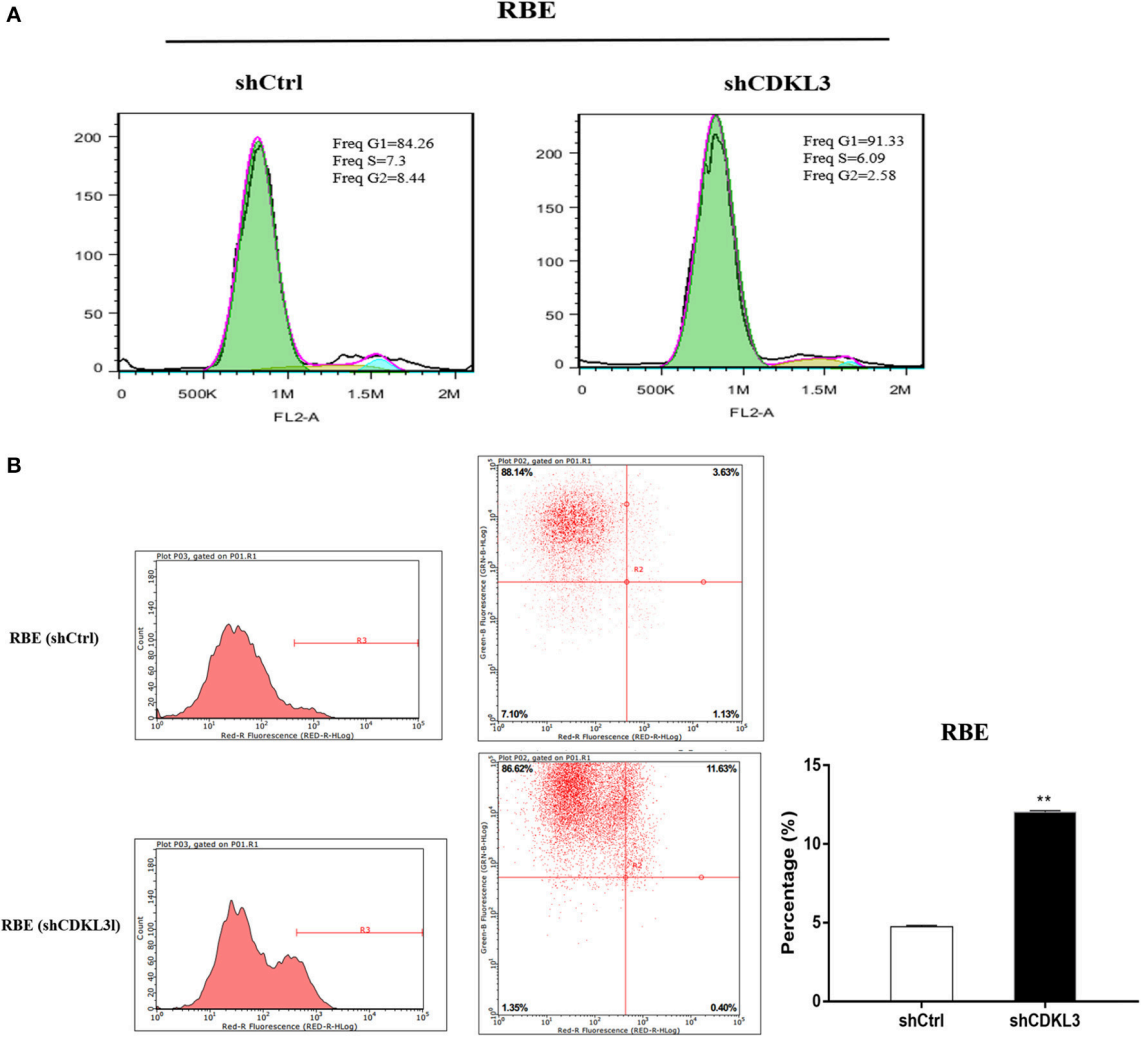
Knockdown of CDKL3 blocks cell cycle progression at G1 phase and induces apoptosis in CCA cells. **(A)** RBE cells transfected with shCDKL3 showed increased percentage of cell population in G0/G1 phase; **(B)** Knockdown of CDKL3 in RBE cells led to a significant increase in apoptosis as compared to the cells without CDKL3 knockdown (12.02% vs. 4.73%, *P* < 0.01). The data represent mean ± SD. *P < 0.05, **P < 0.01, as compared with shCtrl.

Cell cycle blockade is one of the well-established mechanisms for many anticancer agents. As CDKL3 shares similar sequence with CDK3 and the latter encodes a necessary kinase for the G1-S transition in the cell cycle progression of mammalian cells, we thus tested the impact of CDKL3 knockdown on cell cycle progression of CCA cells. As Figure 8 shows, blocking CDKL3 in RBE cells not only increased percentage of cell population in G0/G1 phase (**A**), but also led to a significant induction of apoptosis (**B**).

## Discussion

Curcumol is a pure monomer extracted from the roots of the herbal plant *Rhizoma Curcumae*. Indeed it is a classical small molecular anti-tumor drug which is insoluble in water but easy dissolving in ethyl alcohol and DMSO. Recent studies have shown that curcumol has anti-tumor effect both *in vitro* and *in vivo* (Ning et al., 2016; Huang et al., 2017). However, no studies have been published in its potential effect on CCA. In this study, we attempted different ways to resolve curcumol and eventually we found that curcumol exhibits good solubility in 1% DMSO which has no poisonous effect on cholangiocarcinoma cell lines (Data is not shown). We found that curcumol at the dose of >50 μg/ml could significantly inhibit the proliferation and migration of the CCA cells. The inhibition was in a time-and dose-dependent manner within maximum reference concentration 100 μg/ml (Yu et al., 2016; Wang et al., 2018) but higher than 50 μg/ml, the most significant inhibition concentration of Jurkat cells (Wang et al., 2014). The possible reason may be different cell lines have different sensitivity to curcumol. As aberrantly increased cell proliferation, migration, and invasion are the fundamental biological properties of cancer cells, the significant inhibitory effect of curcumol on these biological functions clearly indicates an anticancer effect of curcumol on CCA cells. Apoptosis is a genetically programmed mode of cell death, and such a mode of cell death is the preferred cell death type in cancer therapies (Fulda, 2014). We observed that curcumol could significantly induce apoptosis in CCA cells, further demonstrating a potential tumoricidal effect of curcumol in CCA.

Cancer cells are generally rapidly dividing, partially due to the impaired cell cycle control as a result of the aberrantly expressed checkpoint proteins. Indeed, uncontrolled cell cycle progression contributes to carcinogenesis and treatment failure (Evan and Vousden, 2001). Hence, we used proteomics and bioinformatics to identify the possible therapeutic target of curcumol. When compared between the control cells and the curcumol-treated RBE cells (100 μg/ml for 48 h), we identified 19 proteins that were differentially expressed at a much higher level in the curcumol-treated RBE cells. Using mass spectrometry, we identified CDKL3 could be a potential molecular target for curcumol.

CDKL3 encodes a polypeptide of 455 amino acids named NKIAMRE, which is localized on the chromosome band 5q31.1 (Midmer et al., 1999; Haq et al., 2001). CDKL3 is a cyclin-dependent kinase-related molecule that is closely related to a subset of Cdc2-related kinases. NKIAMRE is present in the cytoplasm and is thought to be a component of a kinase complex that phosphorylates the C-terminus of RNA polymerase II. NKIAMRE seems to be widely expressed at low levels in all tissues. Published studies have revealed that CDKL3 is related to human cancers (Thompson et al., 2005; Dubos et al., 2008). In this perspective, high expression of CDKL3 was detected in two aggressive types of anaplastic large cell lymphoma (ALCL) tumors when compared to peripheral blood lymphocytes (Thompson et al., 2005). In our present study, we demonstrated a significant up-regulation of CDKL3 in the cytoplasm of CCA cells compared with CCC tissues, suggesting a potential role of CDKL3 in the pathogenesis of CCA. Despite the precise functionality of NKIAMRE remains unclear, sequence similarities between CDKL3 and cyclin-dependent kinase 3 (CDK3), and the presence of several similar motifs on these genes suggest that NKIAMRE is a conserved CDK-related kinase. CDKL3 also has similar sequences with classical MAPK, and NKIAMRE has a potential MAPK activation motif TDY (Thr 158, Asp159 and Tyr 160) in subdomain VIII. Studies have shown that NKIAMRE could not be activated by epidermal growth factor (EGF), 20% FBS, sorbitol, or anisomycin, but it could be stimulated by phorbol 12-myristate 13-acetate (PMA) through phosphorylation of the TDY motif (Yee et al., 2003). This activation pattern is similar to ERK, JNK, and p38, three well-defined MAP kinase subfamilies. It is thus speculated that CDKL3 and its encoded product NKIAMRE may play an important part in the control of cell cycle, cell proliferation just as classical MAPK does. Indeed, our data in the current study show that CDKL3, like its homolog CDK3, has a significant impact on the cell cycle progression in that knockdown of CDKL3 in RBE cells blocked the cell cycle progression from G0/G1 stage to S stage with simultaneous induction of apoptosis. The inhibitory effect of curcumol on cell cycle was further supported by reduced cyclinD1 and CDK4 together with increased expression of P21 and P27. Hence, it is clearly demonstrated that the killing effect of curcumol on CCA cells is related to its inhibitory effect on cell cycle progression.

In summary, curcumol possesses an anticancer effect against CCA through down-regulating CDKL3. Given the marked up-regulation of CDKL3 in CCA, and the strong anticancer effect of curcumol on CCA cells, we speculate that CDKL3 may be a potential therapeutic target for CCA, and in this aspect, curcumol may be a promising anticancer agent. More studies in appropriate animal models are warranted to clarify the biological role of CDKL3 and the anticancer effect of curcumol in the treatment of CCA.

## Author contributions

JZ and GS: Conceived the study and performed the experiment with support from WM, XL, XX, and ZT: Wrote the manuscript and performed the statistical analysis; LW, WF, SZ, and YB: Analyzed and interpreted the results; BB, PY, YL, ZB, and JH: Provided intellectual contribution; LQ: Revised the manuscript and gave final approval of the submitted manuscript. All authors have reviewed and approved the final manuscript.

### Conflict of interest statement

The authors declare that the research was conducted in the absence of any commercial or financial relationships that could be construed as a potential conflict of interest.
